# Frequency of New-Onset Atrial Fibrillation in Post-coronary Artery Bypass Grafting (CABG) Patients in the Cardiac ICU

**DOI:** 10.7759/cureus.83583

**Published:** 2025-05-06

**Authors:** Ujala Akhtar, Hamid Naeem, Sanam Fida, Saddaf Akhtar, Ihtesham Yousuf, Ayesha Zia

**Affiliations:** 1 Medicine, Khyber Teaching Hospital (KTH), Peshawar, PAK; 2 Cardiac ICU, Rehman Medical Institute (RMI), Peshawar, PAK; 3 Cardiac Surgery, Rehman Medical Institute (RMI), Peshawar, PAK; 4 Plastic and Reconstructive Surgery, Khyber Medical College, Peshawar, PAK; 5 General Medicine, Northwest General Hospital, Peshawar, PAK; 6 Cardiology, Khyber Teaching Hospital (KTH), Peshawar, PAK; 7 Internal Medicine, Khyber Teaching Hospital (KTH), Peshawar, PAK; 8 Cardiology, Khyber Medical College, Peshawar, PAK

**Keywords:** arrhythmia, atrial fibrillation, cabg, cardiology, icu

## Abstract

Background: Atrial fibrillation (AF) is a common complication following coronary artery bypass grafting (CABG) and is associated with increased morbidity and prolonged hospital stay. This study aimed to determine the frequency of new-onset atrial fibrillation (NOAF) in post-CABG patients and assess its association with demographic and clinical variables, including comorbidities.

Methods: This descriptive observational study was conducted in the Cardiac ICU of Rehman Medical Institute (RMI) over six months. A total of 101 post-CABG patients admitted to the ICU were included and monitored for the development of AF. Patients with congenital anomalies, a prior history of arrhythmias, or those who did not provide consent were excluded. Data were collected prospectively, including patient demographics, comorbidities, and antiarrhythmic management. Statistical analysis was performed using SPSS version 26 (Armonk, NY: IBM Corp.), with quantitative variables presented as means±standard deviations and qualitative variables as frequencies and percentages.

Results: The frequency of NOAF in post-CABG patients was found to be 13.86% (n=14). The mean age of the study population was 59.62 years (SD=9.81), with a male predominance (69 males, 32 females). Hypertension (HTN) and coronary artery disease (CAD) were the most common comorbidities, affecting 29.7% (n=30) and 20.8% (n=21) of patients, respectively. A trend was observed suggesting that patients with multiple comorbidities had an increased likelihood of developing AF, although this finding was descriptive and not statistically significant. Antiarrhythmic medications were administered in all AF cases, with a variable response.

Conclusion: The incidence of NOAF in post-CABG patients remains significant based on previous researches. Age and pre-existing comorbidities, particularly hypertension and CAD, appear to contribute to AF development. Developing preoperative and intraoperative risk assessment tools may help identify patients at higher risk for post-CABG atrial fibrillation. Future interventional studies can evaluate strategies such as fluid management, early beta-blockers, or anti-inflammatory therapies to reduce the incidence of AF.

## Introduction

Atrial fibrillation (AF) is the most common cardiac arrhythmia following coronary artery bypass graft (CABG) surgery, with an incidence ranging between 20% and 40% [1]. Postoperative AF can be categorized as either transient, where the arrhythmia resolves spontaneously or with treatment during the hospital stay, or persistent, which continues beyond the immediate postoperative period and may require long-term management. AF worsens patient hemodynamic status and increases the risk of embolic events, congestive heart failure, and longer ICU stay [2]. Understanding the nature of postoperative AF, whether transient or persistent, is crucial for developing effective preventive and therapeutic strategies.

The precise mechanism of post-CABG AF is still under investigation; however, it can be due to several factors that can increase the risk of developing atrial fibrillation after CABG surgery. These include advanced age, male gender, a history of heart failure or AF, chronic lung disease (like chronic obstructive pulmonary disease {COPD}), kidney dysfunction, diabetes, obesity, and high blood pressure before surgery. In addition, postoperative factors such as inflammation, fluid overload, longer bypass time, low oxygen levels, and electrolyte imbalances may also contribute to the development of AF [3].

A secondary analysis of the Atrial Fibrillation Suppression Trial II (AFIST) found that patients who developed post-CABG atrial fibrillation received an additional 1.3 L of fluid compared to those who did not. The net fluid balance on the second postoperative day emerged as a significant predictor of post-CABG atrial fibrillation [4]. These findings underscore the importance of careful perioperative fluid management and support its consideration as a potential confounding factor when studying AF incidence post-CABG. Diagnosing AF in the hospital is usually not difficult, as most patients are on continuous monitoring. Initial management should include the correction of predisposing factors such as hypoxemia, electrolyte abnormalities, pain management, and withdrawal of inotropic support. The approach to managing postoperative atrial fibrillation focuses on controlling the ventricular rate, with the ultimate objective being the restoration of sinus rhythm [5].

## Materials and methods

This study was a descriptive observational study conducted in the Cardiac ICU of Rehman Medical Institute (RMI) to assess the frequency and risk factors associated with new-onset atrial fibrillation (AF) in post-coronary artery bypass grafting (CABG) patients.

Patients admitted to the Cardiac ICU following CABG were monitored using electrocardiography for the development of new-onset atrial fibrillation (AF) and were included in the study. Fluid management was standardized across all patients using the 4-2-1 rule based on body weight to minimize variability in fluid administration. All participants provided informed consent before data collection.

A dedicated team of doctors stationed in the Cardiac ICU monitored and recorded data on post-CABG patients developing AF. Post-CABG AF was defined as an absence of P waves and irregularly irregular rhythm on ECG. The team tracked patients through their medical records and continuous ECG monitoring, documenting their response to antiarrhythmic drugs, such as reverting to sinus rhythm. The study team followed each case throughout their ICU stay for two to three days, analyzing ECG data and medical responses. Based on the collected data, statistical analysis was performed, and conclusions were drawn regarding the incidence and risk factors of AF in post-CABG patients.

Study design, setting, population, duration

This study was a descriptive observational study and involved all patients who met the case definition of atrial fibrillation. The study will be conducted in the Cardiac ICU of Rehman Medical Institute. The post-CABG population giving consent, admitted in the Cardiac ICU, fulfilling the inclusion criteria will be recruited for the study. The study will be conducted six months after approval of the synopsis.

Data analysis

Data were analysed using SPSS version 26 (Armonk, NY: IBM Corp.) (for Windows) and statistical analysis. Quantitative variables, such as age and weight, were reported as means and standard deviations (SD). Qualitative variables, such as gender, comorbidities, and medication responses, were presented as frequencies and percentages.

Ethical considerations

Ethical approval was obtained from the Institutional Research and Ethical Board (IREB) of the RMC. Confidentiality of all patient data was strictly maintained. Patients were informed of their right to refuse participation or withdraw from the study at any point without any consequences. No personal or intrusive questions were asked, and all ethical guidelines were strictly followed.

## Results

**Figure 1:**
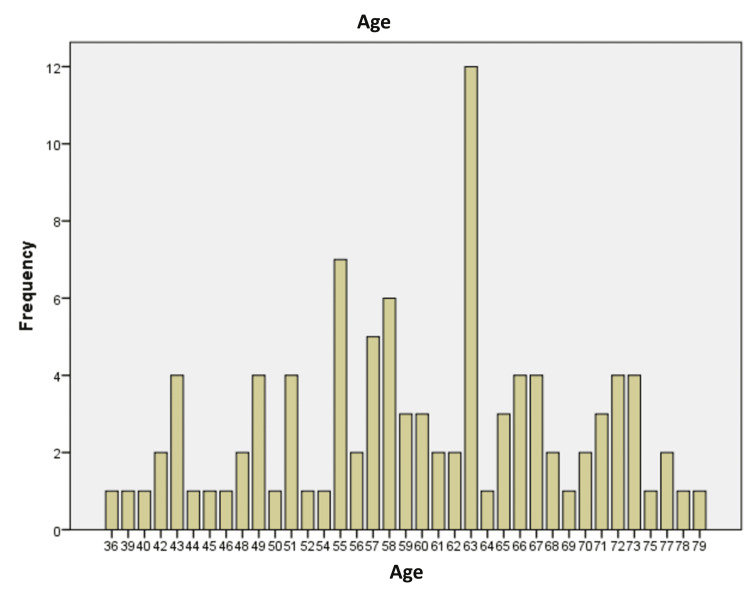
Age distribution of post-CABG patients. CABG: coronary artery bypass grafting

In our study, the frequency of new-onset atrial fibrillation (AF) following CABG was found to be 13.86% (n=14). The study included a total of 101 patients who underwent coronary artery bypass grafting (CABG), with a mean age of 59.62 years (SD=9.81 years). The age range of patients spanned from 36 to 79 years, indicating a predominantly middle-aged and older patient population (Figure 1).

The mean age of patients diagnosed with AF (61 years) was slightly higher than those without AF (59 years), but the difference was not statistically significant (p>0.05) (Table 1). This demographic highlights the importance of monitoring the effects of CABG on aging individuals who may have multiple comorbidities. Regarding the weight of the patients, the mean weight was 76.89 kg (SD=13.98 kg), with a range from 43 kg to 110 kg, demonstrating a diverse body weight distribution in the study sample. Patients diagnosed in the cardiac intensive care unit (CICU) had a higher mean weight (85 kg) compared to those without AF (76 kg), but this difference was not statistically significant (p>0.05).

**Table 1:** Mean age at diagnosis of AF. AF: atrial fibrillation

Age			
Diagnose with AF	Mean	n	Std. deviation
No	59.41	87	10.054
Yes	60.80	14	8.470
Total	59.62	101	9.806

The gender distribution revealed 32 female patients and 69 male patients. Pearson's chi-square test did not show a statistically significant association between gender and AF occurrence (p=0.651). A crosstabulation of gender and comorbidities showed a variety of associated conditions, including hypertension (HTN), diabetes mellitus (DM), coronary artery disease (CAD), and others, with the most common comorbidity combinations occurring in male patients.

Among the patients who developed atrial fibrillation (AF) postoperatively, the average number of AF episodes was 1.5 (SD=0.71), indicating that most cases involved one or two episodes. This further underscores the importance of postoperative monitoring, especially given the high incidence of atrial fibrillation in CABG patients.

The comorbidity distribution among the patients was as follows: female patients predominantly presented with hypertension (HTN) and a variety of comorbidity combinations, such as HTN, CAD, and DM. The most frequent combinations in female patients were HTN (five occurrences) and HTN, CAD (seven occurrences). Male patients showed a broader range of comorbidities, with the most frequent combinations being HTN, CAD, and DM, as well as HTN and CAD. Male patients demonstrated a higher prevalence of multiple comorbidities compared to females, with 69 male patients represented in the study.

When comparing comorbidities between patients with and without atrial fibrillation (AF) post-CABG, it was observed that all patients diagnosed with AF had at least one comorbidity. The most common comorbidity in the AF group was a combination of diabetes, hypertension, and coronary artery disease (DM, HTN, CAD). In contrast, a small number of patients without AF had no comorbidities at all. Other conditions like chronic kidney disease (CKD), asthma, and isolated hypertension were only present in the non-AF group. This suggests a stronger link between multiple cardiovascular comorbidities and the development of AF following CABG surgery.

In total, comorbidities such as hypertension (HTN), diabetes (DM), coronary artery disease (CAD), and their combinations were prevalent across both genders, reinforcing the need for individualized postoperative care strategies for CABG patients, especially those with multiple underlying conditions (Table 2).

**Table 2:** Mean values of variables or descriptive statistics of variables. CAD: coronary artery disease; CKD: chronic kidney disease; DM: diabetes mellitus; HTN: hypertension

Summary of the data		Mean	Count	Column valid, n%
Age		60	-	-
Gender	Female	-	32	31.7%
	Male	-	69	68.3%
Weight		77	-	-
Comorbidities	No comorbidities	-	5	5.0%
	Asthmatic	-	1	1.0%
	CAD	-	19	18.8%
	CKD	-	1	1.0%
	DM	-	3	3.0%
	DM, CAD	-	4	4.0%
	DM, HTN, CAD	-	30	29.7%
	Dyslipidemia, CAD	-	1	1.0%
	HTN	-	10	9.9%
	HTN, CAD	-	21	20.8%
	HTN, DM	-	3	3.0%
	HTN, DM, CAD, CKD	-	1	1.0%
	HTN, DM, CAD	-	1	1.0%
	HTN, DM, hypothyroid	-	1	1.0%
Hx of atrial fibrillation	No	-	101	100.0%
Cardiac monitoring	Yes	-	87	86.1%
	Yes	-	14	13.9%
Diagnose in CICU	No	-	86	85.1%
	Yes	-	14	14.9%
Antiarrhythmic medication received	No	-	87	86.1%
	Yes	-	14	13.9%

These findings emphasize the importance of tailored interventions in older patients with significant comorbidities, who are at a higher risk of complications following CABG surgery. The results also highlight the necessity for vigilant monitoring of atrial fibrillation episodes and other comorbidities in the postoperative period.

## Discussion

This study found that the frequency of atrial fibrillation (AF) following coronary artery bypass grafting (CABG) was 13.86%, which aligns with some previous studies while differing from others. Rajput et al. reported an AF incidence of 10% post-CABG [6]. However, Khan et al. observed a significantly higher frequency of 49% [7]. These discrepancies may be attributed to differences in patient demographics, surgical techniques, perioperative management, and study methodologies. Despite these variations, AF remains a well-recognized complication of CABG, contributing to increased morbidity and prolonged hospitalization.

While previous studies and our study indicate age as a potential risk factor, this association was not statistically significant [8,9]. The mean age of our study population was 60 years, reinforcing the understanding that older patients are at greater risk due to age-related structural and electrical remodeling of the atria. Increased atrial fibrosis, inflammatory responses, and heightened sympathetic activation in elderly patients contribute to a higher likelihood of developing AF postoperatively [10]. Given that the prevalence of AF increases with age, strategies for early identification and management of high-risk patients are essential.

Among patients who develop atrial fibrillation, males were predominant with a frequency of 68.3%, which is similar to previous studies [11,12]. This may be due to the higher burden of coronary artery disease in males, along with other contributing cardiovascular risk factors. Further research is needed to determine whether sex-specific factors influence AF risk following CABG.

In the present study, patients who developed postoperative atrial fibrillation (AF) had a slightly higher mean weight compared to those who did not develop AF. Although the difference was not statistically significant, this observation may suggest that higher body weight could play a role in increasing the risk of AF, as supported by the literature [13].

Comorbidities also played a crucial role in the development of AF following CABG. The most frequently observed comorbid condition was hypertension, present in 42.5% of patients, followed by coronary artery disease (CAD) and diabetes mellitus (DM). Patients with multiple comorbidities, such as those with a combination of hypertension, diabetes, and CAD, were at an even higher risk of developing AF [14]. These conditions contribute to AF by increasing cardiac workload, promoting atrial dilation, and triggering inflammatory and oxidative stress pathways. Chronic kidney disease (CKD), dyslipidemia, and hypothyroidism were also observed in a smaller subset of patients, further emphasizing the complex interplay between systemic diseases and postoperative cardiac arrhythmias.

Although fluid overload is a known confounding factor in the development of post-CABG atrial fibrillation, fluid management in the present study was standardized for all patients. Each patient received fluid replacement according to the 4-2-1 formula based on body weight, which helped minimize variability and reduce the potential bias associated with fluid imbalance. This consistency in fluid administration enhances the reliability of our findings related to atrial fibrillation and reduces the impact of this confounder [15].

This study has several limitations that should be considered when interpreting the findings. First, the relatively small sample size of 101 post-CABG patients may limit the generalizability of the results to a broader population. Second, the study was conducted at a single center, which may introduce institutional biases in patient management and outcomes. Third, the observational nature of the study restricts the ability to establish causality between risk factors and the development of atrial fibrillation. Additionally, potential confounding variables, such as electrolyte disturbances and variations in surgical techniques, were not extensively controlled. Lastly, follow-up was limited to the ICU stay, preventing an assessment of long-term arrhythmic outcomes after discharge. Future multi-center studies with larger sample sizes and extended follow-up periods are needed to validate and expand upon these findings.

## Conclusions

In conclusion, our study found that atrial fibrillation (AF) occurred in 13.86% of patients following coronary artery bypass grafting (CABG). Age appeared to be a contributing factor, as older patients showed a higher tendency to develop AF. Additionally, comorbidities such as hypertension, coronary artery disease, and diabetes mellitus were commonly observed among patients with AF, suggesting a possible association with increased risk of postoperative AF. Male predominance was also observed.

The presence of multiple comorbid conditions underscores the need for preoperative risk assessment and targeted postoperative monitoring. Developing preoperative and intraoperative risk assessment tools may help identify patients at higher risk for post-CABG atrial fibrillation. Future interventional studies can evaluate strategies such as fluid management, early beta-blockers or amiodarone, or anti-inflammatory therapies to reduce the incidence of AF.
